# Dual-omics analysis of key biomarkers in T cell ubiquitination of rheumatoid arthritis blood and synovial tissue, validated by two-sample Mendelian randomization and qPCR

**DOI:** 10.3389/fimmu.2026.1764990

**Published:** 2026-03-02

**Authors:** Danting He, Haitao Zhao, Jinghong Meng

**Affiliations:** 1 Department of Rheumatology and Immunology, The Third Hospital of Hebei Medical University, Shijiazhuang, Hebei, China; 2 Department of Foot Surgery, The Third Hospital of Hebei Medical University, Shijiazhuang, Hebei, China

**Keywords:** machine learning, rheumatoid arthritis, single-cell, T cell ubiquitination, two-sample Mendelian randomization

## Abstract

**Objectives:**

Rheumatoid arthritis (RA) is a chronic autoimmune disease characterized by synovial inflammation and joint destruction. Abnormal T-cell ubiquitination has been implicated in RA pathogenesis, yet its molecular mechanisms remain unclear.

**Methods:**

Transcriptomic data from RA blood and synovial tissue were analyzed to identify differentially expressed genes (DEGs). Ubiquitination-related module genes were obtained using weighted gene co-expression network analysis (WGCNA), and their overlap with DEGs yielded blood-synovial ubiquitination-related genes (BS-UGs). Single-cell datasets were used to extract T-cell marker genes, and intersection analysis identified T-cell-specific ubiquitination genes (BS-TUGs). Machine learning algorithms (SVM-RFE and Boruta) screened key BS-TUGs. Immune infiltration, transcription factor (TF) regulation, and master regulators were explored. Finally, two-sample Mendelian randomization (MR) was performed to assess causal relationships between key genes and RA.

**Results:**

A total of 521 BS-UGs and 21 candidate BS-TUGs were identified, from which six key genes (DOCK10, DGKA, NOP58, JAK3, GCC2, ANO9) were selected. These genes exhibited significant immune-cell correlations and were regulated by multiple TFs. MR analysis demonstrated a positive causal association between *NOP58* (OR = 1.074, p = 0.001) and RA, and a negative association between *GCC2* (OR = 0.928, p < 0.001) and RA, without heterogeneity or pleiotropy.

**Conclusion:**

Integrative dual-omics and MR analyses identified key ubiquitination-related T-cell genes driving RA pathogenesis. *NOP58* and *GCC2* represent potential causal biomarkers and therapeutic targets, offering novel insights into immune regulation and precision intervention in RA.

## Highlights

- Dual-omics integration identified key ubiquitination-related T-cell genes driving rheumatoid arthritis pathogenesis.
- NOP58 and GCC2 showed causal associations with rheumatoid arthritis in Mendelian randomization analysis.
- These findings provide potential biomarkers and therapeutic targets for precision diagnosis and treatment of rheumatoid arthritis.

## 1 Introduction

Rheumatoid arthritis (RA) is a chronic autoimmune disease characterized by erosive synovial inflammation, progressive cartilage and bone destruction, and eventual joint deformity and disability. In advanced stages, extra-articular organs such as the heart and lungs may also be affected (1). The pathogenesis of RA involves a complex interplay of genetic susceptibility (e.g., HLA-DR4), environmental triggers (e.g., smoking, infections), and immune dysregulation (2–4). Although conventional synthetic and biologic DMARDs effectively control inflammation and delay progression, they do not achieve permanent remission and vary in efficacy and tolerability (5–7). Therefore, identifying novel biomarkers with high specificity and mechanistic significance is essential for improving early diagnosis and personalized treatment.

Ubiquitination, a post-translational modification mediated by ubiquitin-activating enzymes (E1), conjugating enzymes (E2), and ligases (E3), regulates protein degradation, localization, and signaling (8, 9). It plays crucial roles in immune regulation, cell cycle control, and inflammation. Abnormal ubiquitination has been implicated in the pathogenesis of autoimmune diseases, including RA.

T cells play a central role in immune responses by recognizing antigens and differentiating into effector subsets (Th1, Th17) or regulatory subsets (Tregs). Their activation and differentiation depend on metabolic pathways that supply energy and biosynthetic precursors. According to Cornelia M. Weyand et al. (10), the decades-long pathophysiology of RA can be divided into three checkpoints, with T-cell abnormalities contributing at each stage. At checkpoint 1, T cells lose systemic tolerance to post-translationally modified self-antigens such as citrullinated peptides, generating autoantibodies. At checkpoint 2, DNA-repair defects (e.g., ATM or MRE11A insufficiency) and metabolic reprogramming (reduced glycolysis, mitochondrial dysfunction) drive short-lived effector phenotypes. At checkpoint 3, activated T cells interact with synovial fibroblasts and macrophages to sustain inflammation and joint destruction. RA T cells exhibit marked metabolic imbalance, particularly hyperactivation of the mTOR/AMPK axis, promoting cytokine secretion and Th1/Th17 differentiation (11). Tregs, crucial for immune tolerance, are reduced in number and functionally impaired in RA, correlating with disease activity (12). Th17/Treg imbalance is further modulated by dysregulated mTOR/Foxp3, TGF-β/Smads, JAK-STAT, and NF-κB signaling pathways (13).

Ubiquitination plays a regulatory role in negative selection during thymocyte development. For instance, the linear ubiquitin chain assembly complex (LUBAC) complex promotes the maturation of late thymocytes through linear ubiquitination. Deficiency of this complex leads to an inability to eliminate autoreactive T cells. These cells then enter the periphery and trigger autoimmune reactions (14, 15). A similar mechanism may operate in RA, where loss of CD4+ T cell tolerance to self-antigens, such as citrullinated proteins. TRAF3 inhibits IL-2R-JAK-STAT5 signaling by recruiting the phosphatase TCPTP, thereby regulating Treg cell development (16). Abnormal TRAF3 function in RA may reduce Treg cells generation while promoting the proliferation of effector T cells. The ubiquitinated state of RIPK1 regulates NLRP3 inflammasome assembly. In RA synovium, abnormal RIPK1 ubiquitination leads to excessive inflammasome activation and the release of IL-1β and IL-18, which amplifies joint inflammation (17, 18).

T cell ubiquitination regulates T cell activation, differentiation, apoptosis, and immune tolerance (19). Disruption of this process contributes to the onset and progression of autoimmune diseases, including RA. Targeting ubiquitination-related signaling molecules such as TRAF6, A20, and Cbl-b has emerged as a promising strategy for therapeutic intervention in RA (20, 21). The pivotal role of ubiquitination-associated genes in T-cell dysfunction highlights their potential as both mechanistic drivers and diagnostic biomarkers in RA.

Compared with previous hypothesis-driven studies focusing on known ubiquitination regulators, our approach integrates transcriptomics, single-cell analysis, and genetic causality inference to identify previously unrecognized T cell–related ubiquitination genes in RA. In the current work, we conducted a comprehensive integrative analysis combining transcriptomic and single-cell sequencing data from RA patients to identify genes associated with T-cell ubiquitination. Through machine learning algorithms, we prioritized key genes. The regulatory mechanisms of these key genes were systematically investigated through immunophenotyping analysis and transcription factor (TF) regulatory network construction. Furthermore, we performed two-sample Mendelian randomization (MR) analysis to establish causal relationships between key genes and RA pathogenesis, which contributes actively to the further study of RA.

## 2 Materials and methods

### 2.1 Data source

The datasets used in this study were downloaded from the publicly available GEO database (https://www.ncbi.nlm.nih.gov/geo/). The GSE89408 dataset (152 RA and 28 control, synovial tissue) and the GSE56649 dataset (13 RA and 9 control, blood) were downloaded from GEO database. Performed single-cell level analysis using GSE243917 dataset (11 RA, synovial tissue) and GSE235508 dataset (1 RA, blood). Additionally, genes related to ubiquitination refers to the published literature (22), and the complete gene list is provided in **Supplementary Table S1**.

### 2.2 Identification of BS-UGs involved in RA

Differential expression analyses were performed separately for synovial tissue (GSE89408) and peripheral blood (GSE56649) to identify genes differentially expressed between RA and control samples. The analyses were conducted using the limma framework, and multiple-testing correction was applied using the Benjamini–Hochberg method. Genes with a false discovery rate (FDR) < 0.05 and |log2 fold change| > 0.5 were defined as differentially expressed genes (DEGs). For sensitivity assessment, DEG results obtained under nominal p-value thresholds were additionally examined and are reported in the **Supplementary Materials**. To quantify ubiquitination activity in each RA blood sample, we calculated a ubiquitination score using single-sample Gene Set Enrichment Analysis (ssGSEA), implemented via the R package GSVA (version 1.42.0). The reference gene set for ubiquitination was curated from a previously published study (22), and includes E1 ubiquitin-activating enzymes, E2 conjugating enzymes, E3 ligases, and deubiquitinating enzymes (DUBs). This score reflects the relative enrichment of ubiquitination-related genes in each sample.

The WGCNA was then performed on the GSE56649 dataset to identify gene modules associated with ubiquitination score. Prior to network construction, sample quality was assessed by hierarchical clustering based on Euclidean distance using the hclust function, and no obvious outlier samples were detected. Genes with low variability were filtered to reduce noise before downstream analysis. A co-expression network was constructed using the unsigned network approach implemented in the WGCNA R package. The soft-thresholding power was selected using the pickSoftThreshold function by evaluating scale-free topology criteria across powers from 1 to 20, and a power value of 3 was chosen, corresponding to a scale-free topology fitting index (R^2^) greater than 0.85. The blockwiseModules function was applied with the following parameters: minimum module size = 50, mergeCutHeight = 0.4, reassignThreshold = 0, and Pearson correlation as the similarity measure. This procedure resulted in the identification of multiple co-expression modules. Pearson correlation analysis was performed to calculate the correlation coefficients and corresponding p-values between module eigengenes and the ubiquitination score derived from ssGSEA. Modules showing the strongest positive or negative correlations with the ubiquitination score and statistical significance (p < 0.05) were defined as ubiquitination-associated key modules and selected for downstream analysis. To ensure that the observed module–score associations were not driven by individual samples, the overall sample distribution and module eigengene expression patterns were examined, and no single sample exhibited disproportionate influence on the correlations.

Next, the BS-UGs were obtained by overlapping WGCNA module genes and DEGs, followed by enrichment analysis using gene ontology (GO) and Kyoto encyclopedia of genes and genomes (KEGG). Additionally, protein-protein interaction (PPI) analysis of BS-UGs was performed using STRING (https://cn.string-db.org) database.

### 2.3 Single-cell data processing and analysis

For the single-cell datasets, we downloaded the raw data for quality control and data filtering using the R package Seurat. Cells with 200 < n.features < 4000, n.Count < 10000 and percent.mt < 10% were retained. The data were then normalized and scaled to identify highly variable genes and principal components. Dimensionality reduction of the cells was achieved through principal component analysis (PCA), and clustering was performed using uniform manifold approximation and projection (UMAP) with a resolution of 0.4. Then, we identified marker genes for each cluster and annotated cell types. Furthermore, KEGG pathway enrichment analysis was performed for cells to investigate the functional differences. Additionally, T cells from blood and synovial tissue were subclustered, followed by a pseudo-time analysis of the distinct clusters within T cells using the R package Monocle 2. Using the “FindAllMarkers” method, we applied the criteria avgFC > 0.5 and p < 0.05 to identify marker genes for T cells in both blood and synovial tissue. The genes obtained by taking the intersection of the marker genes of T cell from blood and synovial tissue were defined as BS-TGs. By intersecting BS-TGs and BS-UGs, we identified the candidate BS-TUGs.

### 2.4 Machine learning analysis

Two machine learning algorithms, Boruta and SVM-RFE, were utilized to screen for BS-TUGs. Key BS-TUGs were identified by overlapping the genes obtained from Boruta and SVM-RFE. Subsequently, Spearman correlation analysis was conducted to calculate the correlations among the key BS-TUGs. Moreover, a nomogram for key BS-TUGs was constructed. The accuracy and clinical utility of this nomogram were evaluated using the calibration curve, DCA curve and ROC curve.

### 2.5 Characterization of key BS-TUGs

Based on the expression profiles of key BS-TUGs, we divided the blood samples into high- and low-expression groups. GSEA was performed to investigate the molecular functions of key BS-TUGs, using c2.cp.kegg.v7.5.1.symbols.gmt as the reference gene set. Furthermore, Gene Set Variation Analysis (GSVA) enrichment analysis was conducted to analyze the pathways enriched in low- and high-expression groups. Additionally, the infiltration of 22 immune cells between RA and control samples was compared using CIBERSORT. Following this, a Spearman correlation analysis was conducted between the key BS-TUGs and differential immune cells. Moreover, the expression level variations of BS-TUGs during the pseudotime trajectory of T cells differentiation were visualized.

### 2.6 Construction of transcription factor-feature gene network

The TFs regulating key BS-TUGs were predicted using the miRNet database (http://www.mirnet.ca). Furthermore, we utilized corto to evaluate the master regulators during the transition from normal state to RA. Subsequently, a Spearman correlation analysis was conducted between the key BS-TUGs and the differential master regulators.

### 2.7 Two sample Mendelian randomization study

The datasets for this MR study were obtained from the publicly accessible IEU Open GWAS database (https://gwas.mrcieu.ac.uk/), treating eQTL data of BS-TUGs as the exposures and RA as the outcome. Detailed information for each exposure, including eQTL dataset ID, tissue context (blood-based eQTLs), sample size, ancestry (European), and number of SNPs, is provided in **Supplementary Table S2**. Only cis-eQTLs were selected as instrumental variables, defined as variants located within ±1 Mb of the corresponding gene, in order to reduce the risk of horizontal pleiotropy. Single nucleotide polymorphisms (SNPs) associated with gene expression at a significance threshold of p < 5 × 10–^6^ were retained. Linkage disequilibrium (LD) pruning was applied using a clumping procedure (r^2^ < 0.001, window size = 50 kb) to ensure independence among instruments. Instrument strength was evaluated by calculating F-statistics, and all retained SNPs exhibited F-statistics greater than 10, indicating a low risk of weak instrument bias. Summary statistics for RA were obtained from a European-ancestry GWAS dataset (bbj-a-73, 5,540 cases and 2,843 controls). Harmonization between exposure and outcome datasets was conducted to align effect alleles, and palindromic SNPs with ambiguous strand orientation were removed. SNPs showing direct associations with the outcome were excluded during the harmonization process to further minimize pleiotropic effects. Causal estimates were primarily derived using the inverse variance–weighted (IVW) method. Additional MR approaches, including weighted median, MR-Egger, simple mode, and weighted mode methods, were applied as complementary analyses to assess the consistency of causal estimates. Heterogeneity among instrumental variables was evaluated using Cochran’s Q test, and horizontal pleiotropy was assessed using the MR-Egger intercept test and MR-PRESSO analysis. Leave-one-out analysis and funnel plot inspection were performed to examine the influence of individual SNPs and the symmetry of effect estimates, while Steiger directionality tests were used to confirm the assumed causal direction from gene expression to RA risk. To account for multiple testing across candidate genes, p-values from the IVW analyses were adjusted using the Benjamini–Hochberg false discovery rate (FDR) method. Only associations passing an FDR threshold of < 0.05 were considered statistically significant. Although formal colocalization analysis was not performed, the MR results were interpreted with caution, acknowledging that distinct causal variants underlying eQTL and RA associations cannot be completely excluded.

### 2.8 The qPCR analysis

After obtaining the approval of the Ethics Committee of the Third Hospital of Hebei Medical University, 2ml of peripheral blood was collected from 5 patients with rheumatoid arthritis who visited the Rheumatology and Immunology Department of the Third Hospital of Hebei Medical University from February 2025 to June 2025, and the synovial tissues of the toe joints of 5 patients with rheumatoid arthritis who underwent toe orthopedics in the Foot and Ankle Surgery Department were collected respectively. It is approximately 0.5×0.5cm in size. The population that underwent health check-ups at the Third Hospital of Hebei Medical University during the same period was selected as the control group. Subsequently, the obtained fresh tissues were temporarily stored in liquid nitrogen storage tanks and transferred as soon as possible to be stored in a -80 °C refrigerator for future use. All patients participating in the study gave informed consent and signed the consent form. The inclusion criteria include: 1. All ages between 18 and 80 years old; 2. All enrolled patients met the classification criteria issued by the American College of Rheumatology (ACR) in 1987 and those issued by the ACR/European League Against Rheumatism (EULAR) in 2010.Total RNA was extracted by utilizing a TRIzol kit (Omega, Norcross, GA, USA) and subjected to reverse transcription Real-time PCR was performed with the SYBR green protocol using gene-specific primers. The levels of the target mRNAs were normalized to these of GAPDH to calculate the relative mRNA expression.

### 2.9 Statistical analysis

All computational analyses were conducted using R software (version 4.4.0). Specific packages and versions include Seurat (version 4.1.0) for single-cell analysis, Monocle2 (version 2.22.0) for pseudotime trajectory, WGCNA (version 1.70-3) for co-expression analysis, GSVA (version 1.42.0) and clusterProfiler (version 4.8.1) for functional enrichment, Boruta and e1071 for machine learning, and TwoSampleMR (version 0.5.6) for Mendelian randomization analysis. Cytoscape (version 3.9.1) was used for network visualization. The full R session information, including all loaded packages and versions, has been provided in the **Supplementary Materials** to ensure reproducibility.

## 3 Results

### 3.1 Identification of BS-UGs involved in RA

**Figure 1:**
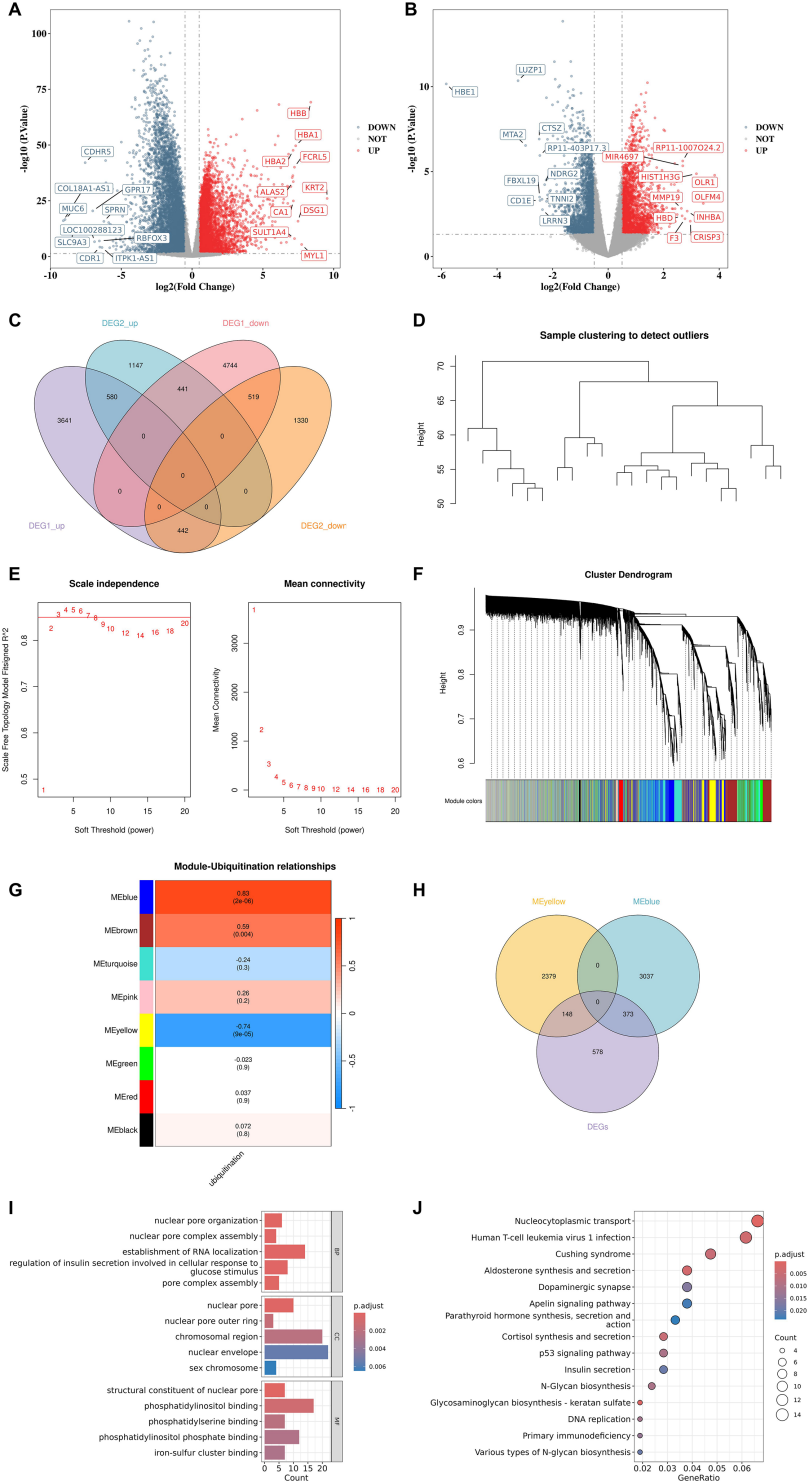
Identification of BS-UGs from synovial and blood transcriptomes. **(A)** Volcano plot of DEGs in synovial tissue showing significantly upregulated and downregulated genes. DEGs were identified using the limma package with thresholds of |log2FC| > 0.5 and FDR-adjusted p < 0.05 (Benjamini–Hochberg correction). **(B)** Volcano plot of DEGs in peripheral blood. **(C)** Venn diagram showing overlap of upregulated and downregulated DEGs between synovial tissue and blood. **(D)** Sample clustering dendrogram showing the absence of outlier samples. **(E)** Scale-free topology fit index and mean connectivity across soft-thresholding powers. **(F)** Gene clustering dendrogram identifying multiple co-expression modules. **(G)** Module–trait heatmap showing correlations between ubiquitination score and gene modules. **(H)** Venn diagram of MEyellow, MEblue and DEGs showing 521 intersecting BS-UGs. **(I)** GO enrichment of BS-UGs performed using clusterProfiler, with significance defined as FDR-adjusted p < 0.05. **(J)** KEGG pathway enrichment of BS-UGs using the same statistical criteria.

A total of 10,367 DEG1s were screened from synovial tissue samples, among which 4,663 genes exhibited an upregulation trend, and 5,704 genes showed a downregulation trend (**Figure 1A**). In blood samples, 4,459 DEG2s were identified (**Figure 1B**), with 2,168 genes upregulated and 2,291 genes downregulated. Subsequently, we obtained 580 DEGs that demonstrated an upregulation trend and 519 DEGs that exhibited a downregulation trend (**Figure 1C**). The Hclust function was utilized to perform cluster analysis on the samples, and a dendrogram of sample clustering was plotted to detect the presence of outlier samples. The result indicated that no outlier samples existed, and all samples were included in the network construction (**Figure 1D**). We set the soft threshold to 3 with the R^2^>0.85 to ensure a scale-free topology of the network (**Figure 1E**). By assessing gene correlation, we constructed a gene hierarchy clustering dendrogram (**Figure 1F**), which allowed us to identify 8 distinct gene modules. Among these modules, ME yellow (2,527 genes) and ME blue (3,410 genes) showed the highest |cor| and significant p-value (**Figure 1G**). A total of 521 BS-UGs were obtained by overlapping 2,527 yellow module genes, 1,099 DEGs, 3,410 blue module genes (**Figure 1H**). The enrichment results from GO confirmed that those genes were associated with establishment of RNA localization, regulation of insulin secretion involved in cellular response to glucose stimulus, nuclear envelope, chromosomal region, phosphatidylinositol binding, phosphatidylinositol phosphate binding (**Figure 1I**). The enrichment results from KEGG confirmed that these genes were associated with nucleocytoplasmic transport, human T-cell leukemia virus 1 infection, Cushing syndrome, dopaminergic synapse, p53 signaling pathway, apelin signaling pathway (**Figure 1J**). The PPI network of BS-UGs was constructed based on interactions with a score > 0.7 (**Supplementary Figure S1**).

### 3.2 Single-cell analysis identifies BS-TUGs

**Figure 2:**
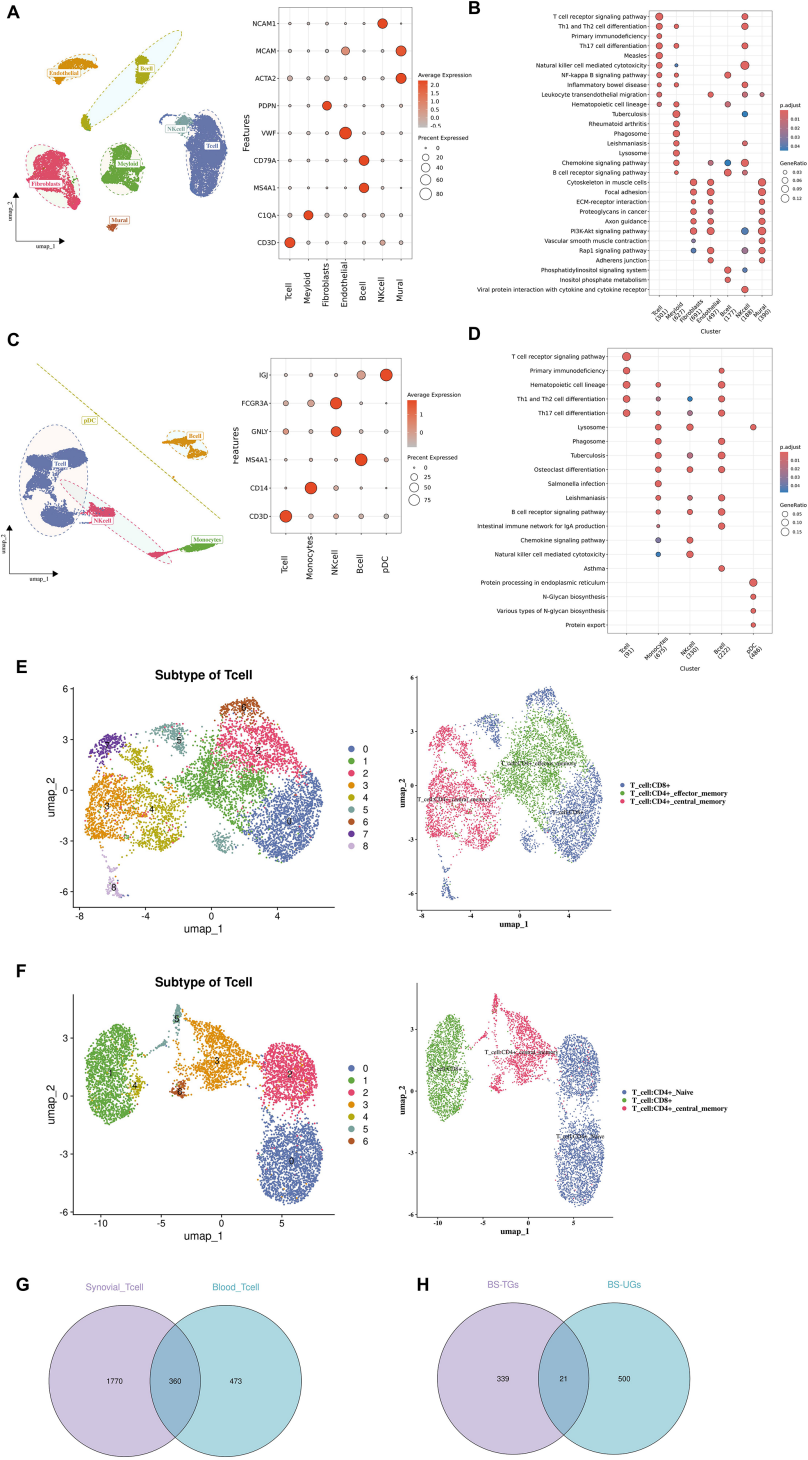
Single-cell analysis identifying BS-TUGs. **(A)** UMAP visualization of synovial cells annotated into major cell types. **(B)** KEGG enrichment analysis of synovial cell clusters, conducted using clusterProfiler with FDR-adjusted p < 0.05. **(C)** UMAP visualization of blood cells annotated into major immune cell types. **(D)** KEGG enrichment of blood cell clusters. **(E)** Reclustering of synovial T cells into nine subpopulations. **(F)** Pseudotime trajectory of synovial T cells showing 17 differentiation states. **(G)** Venn diagram showing overlap of synovial and blood T-cell marker genes (BS-TGs). **(H)** Intersection of 360 BS-TGs and 521 BS-UGs yielding 21 BS-TUGs.

After quality control, 17,110 synovial tissue cells and 9,659 blood cells were retained for downstream analyses (**Supplementary Figure S2**, **Supplementary Figure S3**). By normalization, the 2,000 most variable genes were extracted for PCA, in which the top 20 PCs were selected by the Elbowplot feature. Cells were non-linear reduced dimensionally by UMAP, and both synovial tissue and blood cells were divided into 14 clusters (**Supplementary Figure S4**, **Supplementary Figure S5**). The synovial tissue cells were then annotated by known markers into T cells, myeloid cells, B cells, endothelial cells, fibroblasts, mural cells, NK cells (**Figure 2A**). The KEGG results indicated that T cells were enriched in T cell receptor signaling pathway; myeloid cells were enriched in tuberculosis, phagosome, lysosome and chemokine signaling pathway; fibroblasts were enriched in cytoskeleton in muscle cells, focal adhesion and PI3K-Akt signaling pathway; endothelial cells were enriched in leukocyte transendothelial migration and Rap1 signaling pathway; B cells were enriched in B cell receptor signaling pathway, NF-kappa B signaling pathway and phosphatidylinositol signaling pathway; NK cells were enriched in natural killer cell mediated cytotoxicity and viral protein interaction with cytokine and cytokine receptor; mural cells were enriched in cytoskeleton in muscle cells, focal adhesion and PI3K-Akt signaling pathway (**Figure 2B**). And blood cells were then annotated into: T cells, monocytes, B cells, NK cells, pDCs (**Figure 2C**). The KEGG results indicated that T cells were enriched in T cell receptor signaling pathway, primary immunodeficiency; monocytes were enriched in lysosome, phagosome, tuberculosis, salmonella infection; NK cells were enriched in th1 and th2 cell differentiation, chemkine signaling pathway; B cells were hematopoietic cell lineage, intestinal immune network for IgA production and asthma; pDCs were enriched in protein processing in endoplasmic reticulum, N-glycan biosynthesis, various types of N-glycan biosynthesis (**Figure 2D**). The synovial tissue T cells were further clustered into 9 subpopulations, annotated as: T cell: CD8+, T cell: CD4+effector memory, T cell: CD4+ central memory (**Figure 2E**). Pseudo-time analysis revealed that T cells were divided into 17 states, and state 1 was the root and differentiated to state 10 (**Figure 2F**). Meanwhile, blood T cells were divided into 7 subpopulations, annotated as: T cell: CD4+ naive, T cell: CD8+, T cell: CD4+ central memory (**Supplementary Figure S6**). Pseudo-time analysis revealed that T cells were divided into 7 states, and state 1 was the root and differentiated to states 4 and 6 (**Supplementary Figure S7**). Using FindALLMarkers, 2,130 genes were obtained for synovial T cells and 833 genes for blood T cells. By overlapping these genes, 360 candidate BS-TGs were identified (**Figure 2G**). By intersecting the 360 candidate BS-TGs and 521 candidate BS-UGs, 21 BS-TUGs were obtained (**Figure 2H**).

### 3.3 Machine learning identifies key BS-TUGs in RA

**Figure 3:**
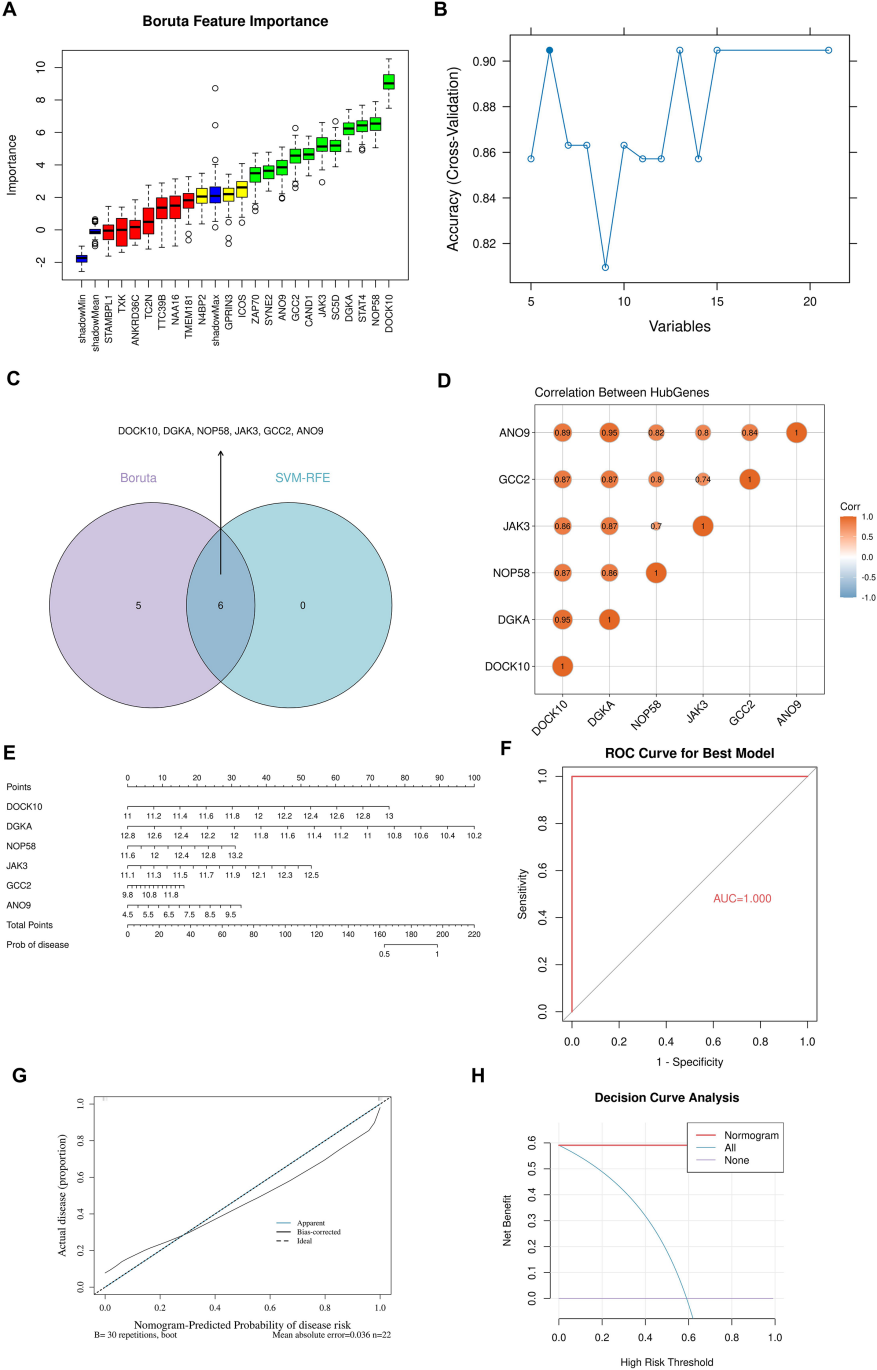
Machine-learning identification and validation of six key BS-TUGs. **(A)** Boruta feature-selection ranking of 21 candidate BS-TUGs. **(B)** SVM-RFE performance curve across different variable numbers. **(C)** Venn diagram showing six overlapping genes selected by Boruta and SVM-RFE. **(D)** Correlation matrix among the six key BS-TUGs. **(E)** Nomogram constructed using the six key BS-TUGs. **(F)** ROC curve demonstrating the discriminative ability of the model. **(G)** Calibration curve assessing agreement between predicted and observed probabilities. **(H)** Decision curve analysis illustrating the net clinical benefit.

To identify key BS-TUGs from 21 candidate BS-TUGs, SVM-RFE and Boruta algorithms were employed. By Boruta, 11 BS-TUGs were screened as important features (**Figure 3A**). Meanwhile, 6 BS-TUGs were identified by the SVM-RFE (**Figure 3B**). Finally, by overlapping genes selected by SVM-RFE and Boruta, 6 BS-TUGs: DOCK10, DGKA, NOP58, JAK3, GCC2, ANO9 were obtained and defined as key BS-TUGs (**Figure 3C**). Moreover, the strong positive correlations were observed among key BS-TUGs (**Figure 3D**). Additionally, the AUC value of the ROC curve was 1.000; the calibration curve lay near the diagonal line, and the DCA outcomes confirmed that the application of this nomogram could yield clinical benefits (**Figures 3E–H**). In summary, this prediction model based on key BS-TUGs demonstrated good performance.

### 3.4 Analysis of the biological characteristics of key BS-TUGs

**Figure 4:**
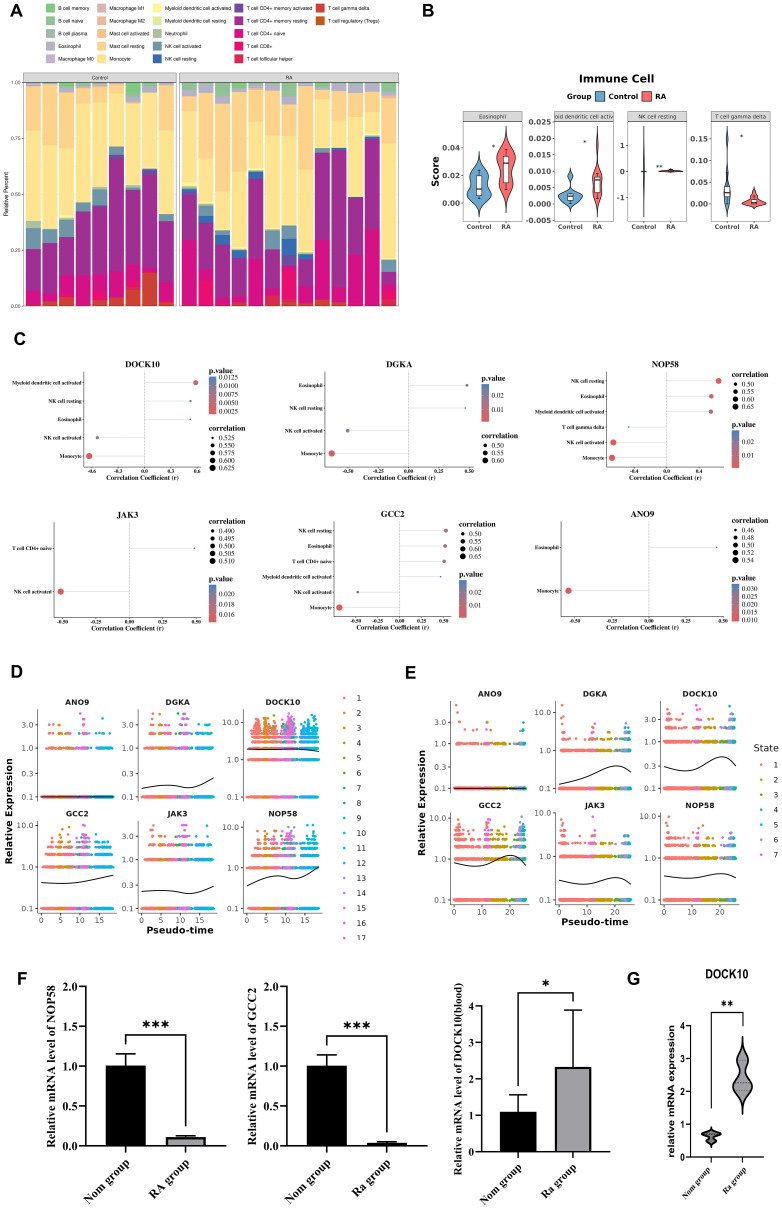
Biological characteristics of key BS-TUGs. **(A)** Relative proportions of 22 immune-cell types in RA and control groups. **(B)** Differential abundance of selected immune cell types between RA and controls assessed using the Wilcoxon rank-sum test with FDR correction. **(C)** Correlations between key BS-TUGs and immune-cell infiltration. **(D)** Expression trends of six key BS-TUGs along synovial T-cell pseudotime. **(E)** Expression trends of key BS-TUGs along blood T-cell pseudotime. **(F)** qPCR validation of NOP58, GCC2, and DOCK10 expression in peripheral blood samples; differences were evaluated using a two-tailed Student’s t-test. **(G)** qPCR validation of DOCK10 expression in RA versus non-RA synovial tissue using the same statistical test. *p < 0.05, **p < 0.01, ***p < 0.001.

To explore the potential mechanisms of key BS-TUGs in regulating RA, we first conducted GSEA analysis. The genes ANO9, DGKA, DOCK10, GCC2 and JAK3 all inhibited antigen processing and presentation, cytokine-receptor interaction, graft versus host disease, leishmania infection and natural killer cell mediated cytotoxicity. As the expression level of NOP58 increased, the activation state was exhibited in spliceosome (**Supplementary Figure S8**). Furthermore, GSVA enrichment analysis revealed that pathways such as aminoacyl tRNA biosynthesis, RNA polymerase, nucleotide excision repair, RNA degradation were enriched in high expression groups; whereas ether lipid metabolism, neuroactive ligand receptor interaction, calcium signaling pathway were enriched in low expression groups (**Supplementary Figure S9**). Next, a comparison of 22 immune cells between the RA and control were performed. The results showed that eosinophil, myeloid dendritic cell activated and NK cell resting were significantly more abundant in RA group, while γδT cell was significantly less abundant (**Figures 4A, B**). Among them, there were strong positive correlations between myeloid dendritic cell activated and DOCK10, NOP58, GCC2; strong positive correlations between eosinophil and DOCK 10, DGKA, NOP58, GCC2, ANO9; and a strong negative correlation between γδT cell and JAK3 (**Figure 4C**). Additionally, the expression levels of DGKA, DOCK10, GCC2, JAK3 and NOP58 underwent alterations during the differentiation of blood T cells (**Figure 4D**). Similarly, the expression of DOCK10, DGKA, GCC2, JAK3 and NOP58 varied with the differentiation of synovial T cells (**Figure 4E**). To experimentally validate these findings, we performed qPCR analysis of DOCK10, NOP58, and GCC2. DOCK10 was significantly upregulated in peripheral blood from RA patients, while NOP58 and GCC2 were significantly downregulated (**Figure 4F**). Furthermore, DOCK10 was also significantly elevated in RA synovial tissue compared to controls (**Figure 4G**). These results provide preliminary experimental support for the transcriptomic and immune correlation findings, reinforcing the potential role of these genes in RA.

### 3.5 Key genes are regulated by multiple transcription factors

**Figure 5:**
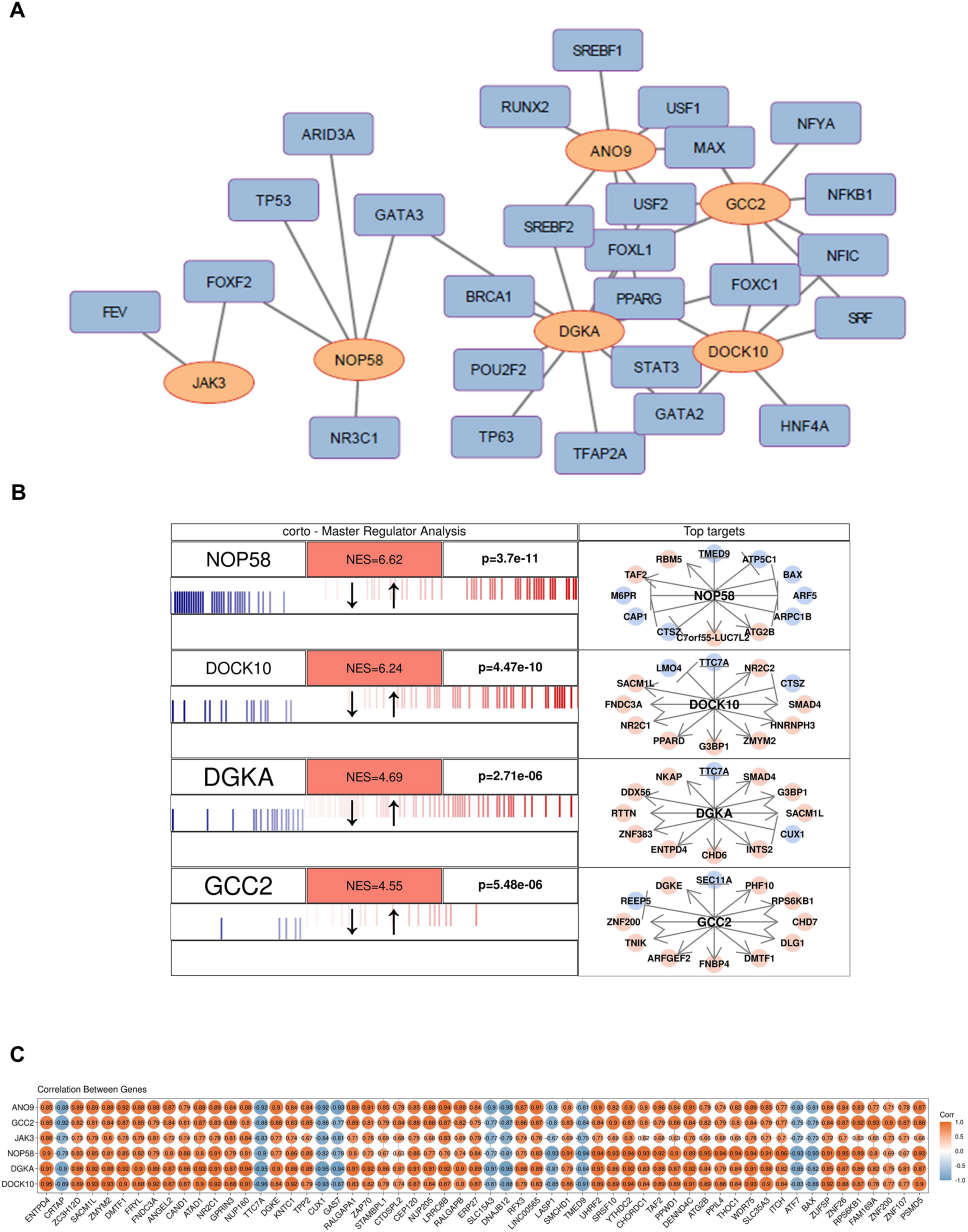
Regulatory network and transcriptional control of key BS-TUGs. **(A)** TF–gene regulatory network predicted for the six key BS-TUGs. **(B)** Master-regulator analysis (MRA) identifying significant regulatory TFs of NOP58, DOCK10, DGKA and GCC2. **(C)** Correlation matrix between TFs and the six key BS-TUGs.

To investigate the regulatory mechanisms of key BS-TUGs in RA, 26 TFs were identified (**Figure 5A**). Among the 6 key BS-TUGs, four genes had master regulators: NOP58 (NES = 6.62, p=3.7e-11), DOCK10 (NES = 6.24, p=4.47e-10), DGKA (NES = 4.69, p=2.71e-06), and GCC2 (NES = 4.55, p=5.48e-06) (**Figure 5B**). By intersecting master regulators and DEGs, we identified 58 differentially expressed master regulators. Among them, CRTAP, TTC7A, CUX1, GAS7, SLC15A3, DNAJB12, LASP1, TMED9, ATF7 and BAX exhibited strong negative correlations with the key BS-TUGs, while the remaining master regulators showed strong positive correlations with key BS-TUGs (**Figure 5C**).

### 3.6 NOP58 and GCC2 demonstrate significant causal association with RA

**Figure 6:**
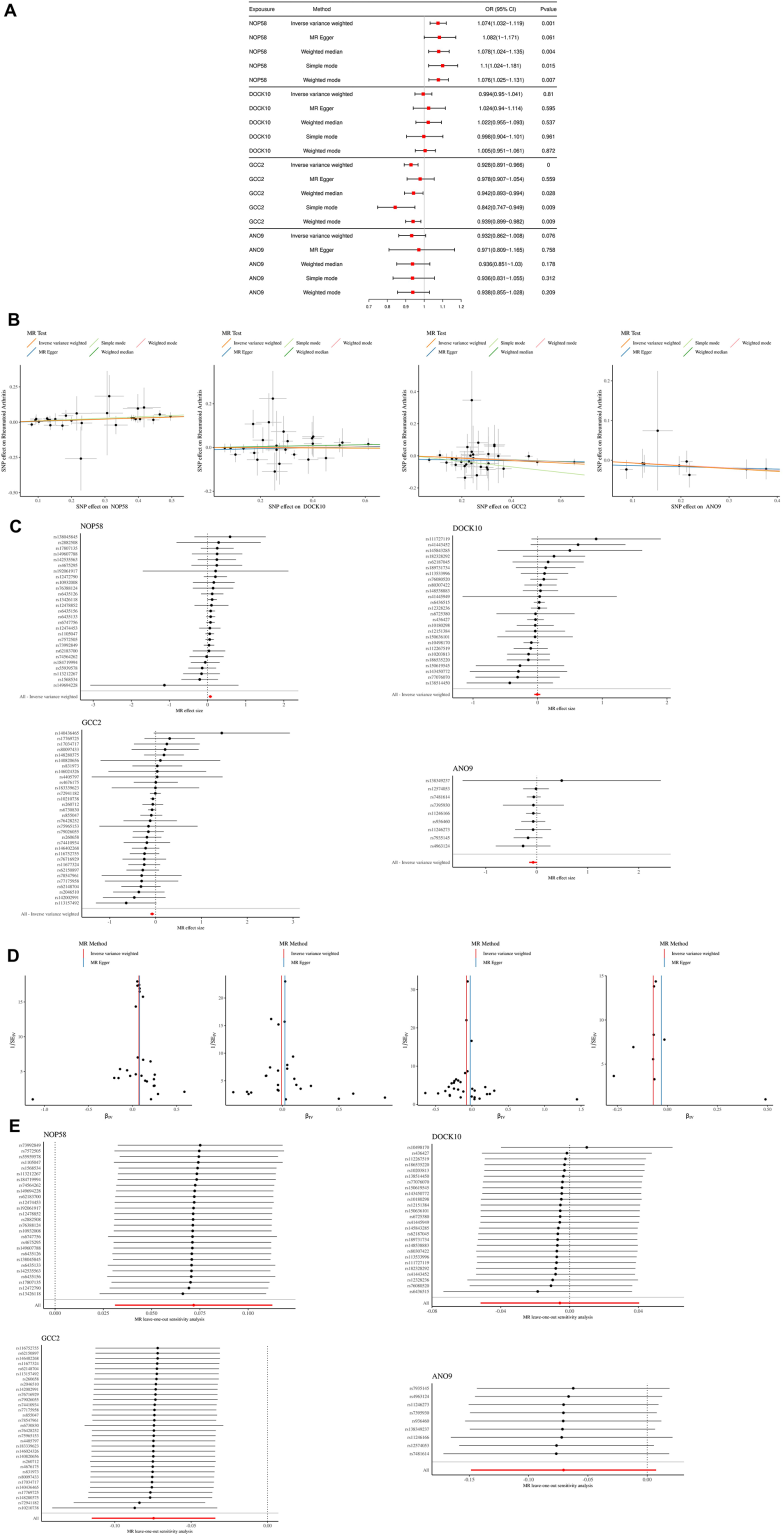
Mendelian randomization analysis of key BS-TUGs and RA. **(A)** Forest plot summarizing causal estimates of NOP58, DOCK10, GCC2 and ANO9 across MR methods. **(B)** Scatter plots showing MR regression lines for each BS-TUG. **(C)** Single-SNP forest plots for MR estimates. **(D)** Funnel plots assessing heterogeneity among SNPs. **(E)** Leave-one-out sensitivity analyses demonstrating robustness of MR estimates.

The data used for MR analysis were shown in. According to the selection criteria of IVs, NOP58, DOCK10, GCC2, ANO9 were used as IVs for RA. As shown in **Figures 6A-C**, NOP58 and GCC2 were found to be associated with RA. Specifically, there was a positive causal association between NOP58 (IVW OR: 1.074, 95% CI: 1.032-1.119, p=0.001) and RA, and a negative causal association between GCC2 (IVW OR: 0.928, 95% CI: 0.891-0.966, p=0) and RA. This result was broadly consistent with those obtained using other MR methods including the weighted median, the MR-Egger, the simple mode and weighted mode. Sensitivity analysis was conducted to verify the reliability of IVW results. The IVW test for heterogeneity showed no heterogeneity in MR analysis results between BS-TUGs and RA (p > 0.05). The pleiotropy test and MR-PRESSO test showed no pleiotropy in MR analysis results (p > 0.05). The funnel plot displayed a symmetric distribution of SNPs, underscoring the relative stability of the results (**Figure 6D**). And the leave-one-out analysis indicted that our MR analyses were not driven by a single SNP (**Figure 6E**). Finally, MR Steiger test found no evidence of reverse causality, and the causal direction was reliable.

## 4 Discussion

In this study, we screened differentially expressed genes from synovial tissue and blood data of RA patients, constructed ubiquitination-related modules by WGCNA, combined with single-cell data to obtain T-cell characteristic genes. We ultimately identified six key genes from 21 candidate BS-TUGs using SVM-RFE and Boruta algorithms. Functional annotation and transcription factor analysis suggested that these genes may be involved in immune dysregulation, and two-sample MR analysis indicated that NOP58 and GCC2 are significantly and causally associated with RA susceptibility.

In autoimmune diseases, dysregulation of T-cell ubiquitination can disrupt immune homeostasis at multiple levels (14). Abnormal ubiquitination acts as a major pathogenic mechanism by finely modulating the molecular networks of immune tolerance and immune activation. This process involves alterations in intrinsic T-cell signaling, weakening of Treg function, and promotion of Th17 polarization, which together reshape the immune microenvironment and aggravate chronic inflammation (23, 24). In RA, defective ubiquitination promotes disease progression by regulating T-cell activation, cytokine secretion, and inflammatory signaling cascades. Importantly, most previous studies in RA have focused on well-characterized ubiquitination regulators, such as TRAF6, Cbl-b, and A20, which are canonical components of the ubiquitination machinery and have defined enzymatic roles. These studies were largely hypothesis-driven and centered on known immune signaling pathways. In contrast, the present study adopts a data-driven, integrative approach to identify ubiquitination-related genes specifically enriched in RA T cells, without restricting the analysis to classical ubiquitination enzymes. Previous studies have shown that ubiquitination of TAK1 and TRAF6 participates in inflammatory amplification in RA, and their aberrant activation leads to sustained release of pro-inflammatory mediators from T cells (25, 26). In contrast, the proteasome inhibitor bortezomib stabilizes IκBα by blocking ubiquitin–proteasome pathway–mediated degradation, thereby reducing NF-κB activation, suppressing pro-inflammatory cytokine release, and improving synovial inflammation in RA (27). Yu (28)et al. found that the E3 ubiquitin ligase Cbl-b was abnormally expressed in RA and its activity was enhanced, and that Cbl-b degraded Foxp3 by ubiquitylation, which regulated the differentiation and function of Treg cells. This led to a decrease in the number of Treg cells or functional defects and weakened their inhibitory effect on autoreactive T cells. In addition, abnormal ubiquitination of TRAF6 inhibits the immunosuppressive function of Treg cells and promotes RA progression (29).

In this study, we mainly screened 6 key genes from 21 candidate genes: DOCK10, DGKA, NOP58, JAK3, GCC2, and ANO9 by SVM-RFE and Boruta algorithms. Notably, among these genes, NOP58 and GCC2 are not canonical E3 ligases or deubiquitinases, nor have they been previously reported in the context of RA or T-cell ubiquitination, suggesting that they may represent noncanonical modulators of ubiquitination-related immune processes. The KEGG enrichment analysis in the present study showed that all six BS-TUGs promotes aberrant recognition of self-antigens by T cells through modulation of MHC molecular expression or APC function, which is directly related to autoimmune mechanisms in RA. Among these genes, JAK3 and NOP58 were significantly enriched in the JAK–STAT signaling pathway, which plays a dominant role in Th17-cell differentiation and pro-inflammatory cytokine secretion in RA. JAK3 has been shown to activate downstream signaling cascades, including the RAS/ERK and PI3K/AKT pathways, through phosphorylation of STAT5, STAT6, and other transcription factors, thereby regulating immune-cell proliferation, differentiation, survival, and functional activation (30). In RA, aberrant activation of the JAK3-STAT5 pathway leads to the over proliferation of Th1 and Th17 cells, which secrete proinflammatory factors such as IL-17 and IFN-γ, and induce the activation of synovial fibroblasts and macrophages, which produce matrix metalloproteinases (MMPs) and prostaglandins, and destroy articular cartilage and bone tissue. pro-inflammatory factors such as IL-17, IFN-γ, etc., inducing activation of synovial fibroblasts and macrophages, production of MMPs and prostaglandins, and destruction of articular cartilage and bone tissues; insufficient or dysregulated JAK3 signaling inhibits the development and function of Treg, weakening its inhibitory ability to effector T cells, leading to loss of immune tolerance; JAK3-STAT6 pathway promotes the differentiation of B cells into plasma cells, production of RF and ACPA, these autoantibodies form immune complexes with antigens, activate the complement system and recruit inflammatory cells to the joint site (31–33). The PPI network in our results shows that JAK3 interacts with Cbl-B, and Cbl-B/E3 ligase inhibits JAK3 phosphorylation through K33 ubiquitination, and the defective ubiquitination results in the sustained activation of the JAK-STAT pathway, driving Th17 cell expansion. The reduced level of JAK3 ubiquitination in synovial tissue was positively correlated with IL-17 secretion, further confirming the important role of JAK3 in RA.

GSEA analysis indicated that NOP58 may be involved in the “antigen processing and presentation” pathway, and could potentially promote Th17 responses via indirect regulation of Notch2, though this is based on enrichment rather than direct evidence. Meanwhile, NFKB1 has been reported to exert an anti-inflammatory function in autoimmune diseases, including RA, by modulating the ubiquitination steps of the NF-κB pathway (34). NFKB1 may activate the NF-κB pathway through the regulation of NOP58 to promote the expression of pro-inflammatory factors such as IL-6 and TNF-α, which is consistent with the speculation, and further illustrates the role of NOP58 (35).

DOCK10, belonging to the DOCK-D subfamily, is expressed in leukocytes and some non-hematopoietic cells, and may be involved in B-cell development and T-cell migration. In addition, DOCK10 may also affect B cells by regulating CD23 expression, but the specific pathway is not clear (36). Members of the DOCK-D subfamily, such as DOCK10 and DOCK11, are known to activate CDC42, thereby participating in cytoskeletal reorganization and immune cell motility (37). These findings collectively suggest that DOCK10 may play a central role in regulating immune-cell interactions within inflamed synovial tissues in RA. It has been demonstrated that DGKA is highly expressed in tumor-infiltrating T lymphocytes, leading to T cell dysfunction, which in turn depletes DAG and inhibits RasGRP1 and PKCθ/NF-κB pathways to help tumors evade immune attack (38, 39). We hypothesize that autoreactive T cells in RA may be under-expressed or functionally deficient in DGKA, leading to over-activation of DAG signaling and sustained secretion of pro-inflammatory factors, such as IL-17 and IFN-γ, to drive synovial inflammation. Our findings also indicate that DGKA may regulate immune-cell infiltration and tissue injury in RA by modulating NK-cell activity, highlighting its involvement in disease progression.

GCC2 was enriched in the cell-cycle checkpoint pathway, supporting the hypothesis that it may inhibit excessive immune-cell proliferation by regulating the T-cell cycle. GCC2 has been found to maintain the integrity of the Golgi, promote the nuclear translocation of epidermal growth factor receptor (EGFR) and activation of the MAPK/ERK signaling pathway, and thus promote the progression of non-small cell lung cancer (NSCLC) (40). Given these functions, GCC2 may influence intracellular trafficking of ubiquitination-related proteins, indirectly modulating immune signaling in RA. While speculative, this hypothesis offers a novel link between Golgi function and T-cell dysregulation in autoimmunity (28). This potential link between ubiquitination machinery and Golgi dynamics offers new insight into T-cell regulation in autoimmune diseases.

ANO9 was enriched in the ubiquitin-mediated protein degradation, suggesting that the abnormalities of ubiquitination were involved in the development of RA through the degradation of T cell-regulated proteins (e.g., cytokine receptor) and caused an imbalance of immunological homeostasis. Experiments showed that knockdown of ANO9 resulted in a complete loss of Ca² signaling in Jurkat T cells and mouse lymphocytes, impaired IL-2 secretion and cell proliferation, and its function was dependent on interaction with the scaffold protein DLG1 (41). In addition, ANO9 is involved in immune tolerance by regulating intracellular vesicle trafficking, and its abnormality may be associated with T cell hyperactivation in autoimmune diseases (41). Our results show that ANO9 is enriched in “ubiquitin-mediated protein degradation”, further confirming that ANO9 is involved in the development of RA through abnormal ubiquitination and consequent degradation of T cell regulatory proteins, leading to an imbalance in immune homeostasis.

Immune infiltration analysis revealed associations between BS-TUGs and specific immune-cell types. Notably, correlations with monocytes, NK cells, and Tregs suggest potential involvement in modulating the RA immune microenvironment. While NOP58 and JAK3 appear to enhance inflammatory cell recruitment, GCC2 may exert protective effects by supporting immune tolerance. These interpretations, although biologically plausible, are correlative and should be considered hypothesis-generating.

MR validated that NOP58 and GCC2 were causally associated with RA, providing genetic evidence that supports their relevance to disease susceptibility rather than reflecting nonspecific immune activation. While these genes may have broader cellular functions, their T cell–specific expression patterns and causal associations with RA suggest a more targeted role in RA pathogenesis. MR verified that NOP58 and GCC2 were causally associated with RA, filling a critical link in the “ubiquitination - T cell - RA” causal chain. NOP58, a ribosome biosynthesis-related gene, may drive RA inflammation by enhancing T cell proliferation and pro-inflammatory cytokine secretion, which correlates with the GSEA enrichment in the “antigen processing and presentation” pathway; and positively correlated with monocyte and NK cell infiltration, suggesting that it drives RA progression by enhancing immune cell activation.GCC2 is mainly involved in cell cycle regulation, and plays a protective role by inhibiting T cell over-activation or promoting the function of Tregs, which is consistent with the GCC2 is mainly involved in cell cycle regulation and plays a protective role by inhibiting T cell overactivation or promoting Tregs function, which is consistent with the positive correlation between GCC2 and Tregs in the immune infiltration analysis, suggesting that GCC2 plays a protective role by maintaining immune tolerance. Therefore, it is inspired that we can target NOP58 to inhibit RA inflammation or enhance the function of GCC2 as a protective therapeutic strategy, and further in-depth mechanistic studies are necessary.

In this study, we systematically revealed the functional roles, regulatory networks, and causal relationships of BS-TUGs in RA through multidimensional biosignature analysis. However, the MR analysis still has certain limitations. Specifically, it was based on GWAS data from European populations, which may limit generalizability across ethnic groups and necessitates validation in other populations. The RA-related mechanisms of ANO9 and DOCK10 remain incompletely understood and require further investigation through cellular and animal experiments. In addition, the single-cell RNA-seq dataset used for peripheral blood (GSE235508) included only one RA sample, which limits the robustness of T cell marker identification in the blood compartment. While this dataset was utilized for preliminary cross-compartment comparison with synovial tissue, we acknowledge that findings derived from this limited sample are exploratory in nature and should be validated in larger, multi-sample scRNA-seq datasets. Further experimental validation is also needed for the MR-prioritized genes; in particular, the functions of NOP58 and GCC2 can be clarified through CRISPR-Cas9–mediated knockdown or overexpression in primary T cells or RA animal models. Additionally, the possibility of synergistic inhibition of transcription factors such as NFKB1 and STAT3 in combination with BS-TUG targeting may offer a promising multi-target strategy to overcome the ceiling effect of monotherapies.

## Data Availability

The datasets presented in this study can be found in online repositories. The names of the repository/repositories and accession number(s) can be found in the article/**Supplementary Material**.

## Supplementary material

Supplementary Figure S1 PPI network of BS-UGs. Protein–protein interaction (PPI) network constructed from 521 BS-UGs based on STRING with an interaction score > 0.7. Nodes represent genes, and edges indicate high-confidence protein interactions. Dense clusters highlight cooperative functional hubs.

Supplementary Figure S2 Quality control of synovial single-cell RNA-seq dataset. **(A)** Violin plot of nFeature_RNA across samples. **(B)** Violin plot of nCount_RNA across samples. **(C)** Violin plot of mitochondrial gene percentage (percent.mt) across samples. These metrics collectively confirm the quality and comparability of synovial cell libraries.

Supplementary Figure S3 Quality control of blood single-cell RNA-seq dataset. **(A)** Violin plot of nFeature_RNA. **(B)** Violin plot of nCount_RNA. **(C)** Violin plot of percent.mt. Quality distributions support reliable downstream single-cell analyses.

Supplementary Figure S4 Synovial single-cell variable gene detection and clustering. **(A)** Scatter plot of standardized variance vs. average expression, highlighting the top 2,000 variable genes. **(B)** JackStraw plot assessing statistical significance of PCs. **(C)** ElbowPlot showing standard deviation across PCs. **(D)** UMAP projection showing 14 clusters of synovial cells.

Supplementary Figure S5 Blood single-cell variable gene detection and clustering. **(A)** Highly variable gene selection in blood cells. **(B)** JackStraw plot showing significance of PCs. **(C)** ElbowPlot identifying major PCs. **(D)** UMAP projection identifying 14 blood cell clusters.

Supplementary Figure S6 Pseudotime analysis of synovial T cells. **(A)** Pseudotime trajectory showing differentiation continuum. **(B)** State assignment of 17 synovial T-cell states. **(C)** Distribution of T-cell subtypes (CD8+, CD4+ effector memory, CD4+ central memory) along trajectory.

Supplementary Figure S7 Pseudotime analysis of blood T cells. **(A)** Pseudotime trajectory showing differentiation progression. **(B)** State assignment of 7 T-cell states. **(C)** Distribution of major blood T-cell subsets (CD4+ naïve, CD8+, CD4+ central memory).

Supplementary Figure S8 GSEA of six key BS-TUGs. GSEA enrichment curves for: **(A)** DOCK10. **(B)** DGKA. **(C)** NOP58. **(D)** JAK3. **(E)** GCC2. **(F)** ANO9. Each panel shows the top KEGG pathways significantly enriched in high- or low-expression samples, including antigen processing and presentation, cytokine–cytokine receptor interaction, natural killer cell–mediated cytotoxicity, and spliceosome.

Supplementary Figure S9 GSVA results for six key BS-TUGs. GSVA comparison of high-expression vs. low-expression groups for: **(A)** DOCK10. **(B)** DGKA. **(C)** NOP58. **(D)** JAK3. **(E)** GCC2. **(F)** ANO9. Bar charts display pathways significantly enriched in each expression group, with pink bars representing pathways activated in high-expression groups and green bars representing pathways activated in low-expression groups.
