# Supplementary figures and images for: Dual-omics analysis of key biomarkers in T cell ubiquitination of rheumatoid arthritis blood and synovial tissue, validated by two-sample Mendelian randomization and qPCR
Source: Front Immunol. 2026 Mar 2;17:1764990. doi: 10.3389/fimmu.2026.1764990 (PMC12989613; doi:10.3389/fimmu.2026.1764990)

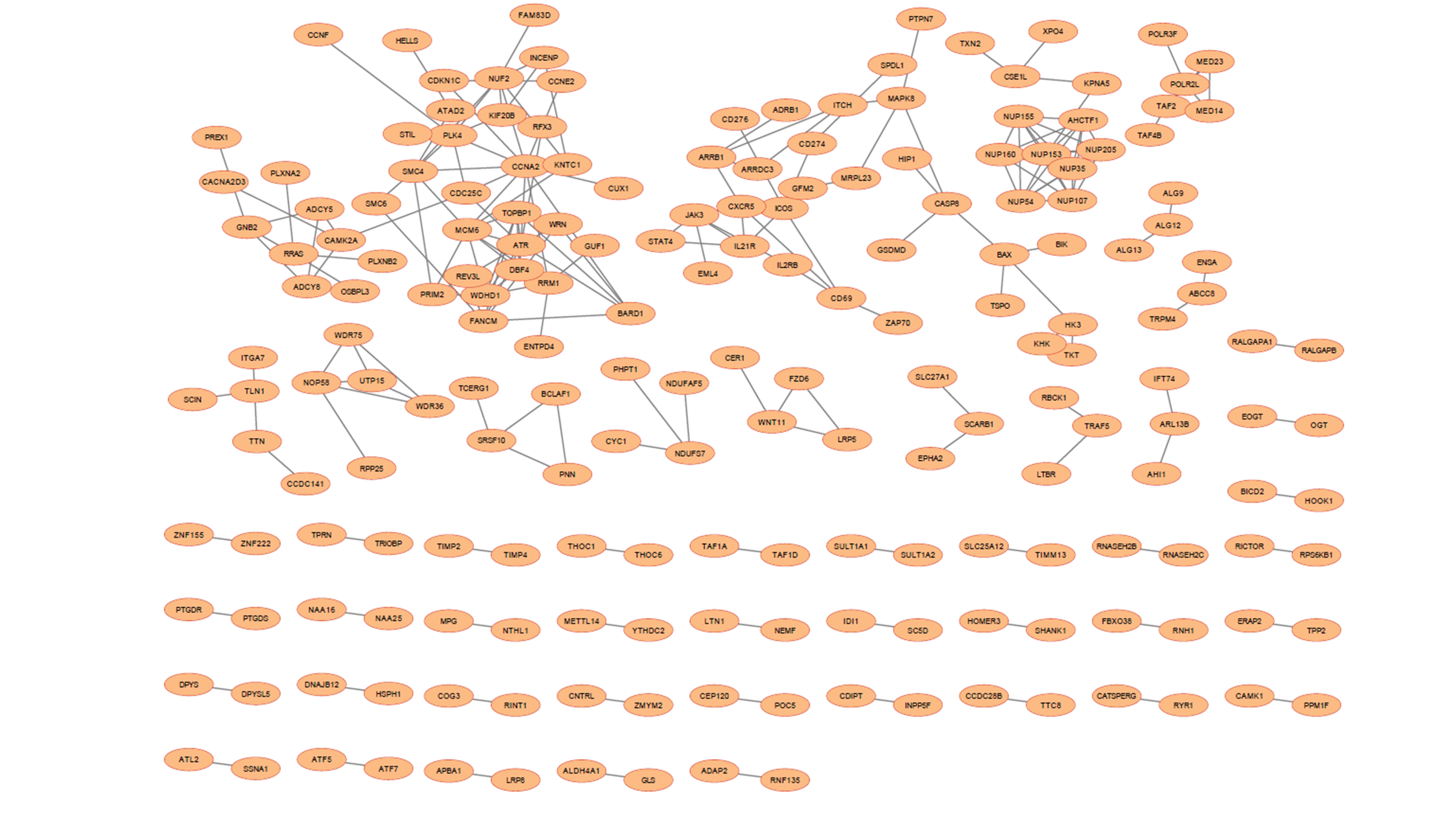

Supplement: Supplementary Figure S1 — PPI network of BS-UGs. Protein–protein interaction (PPI) network constructed from 521 BS-UGs based on STRING with an interaction score > 0.7. Nodes represent genes, and edges indicate high-confidence protein interactions. Dense clusters highlight cooperative functional hubs. [file Image1.jpeg]

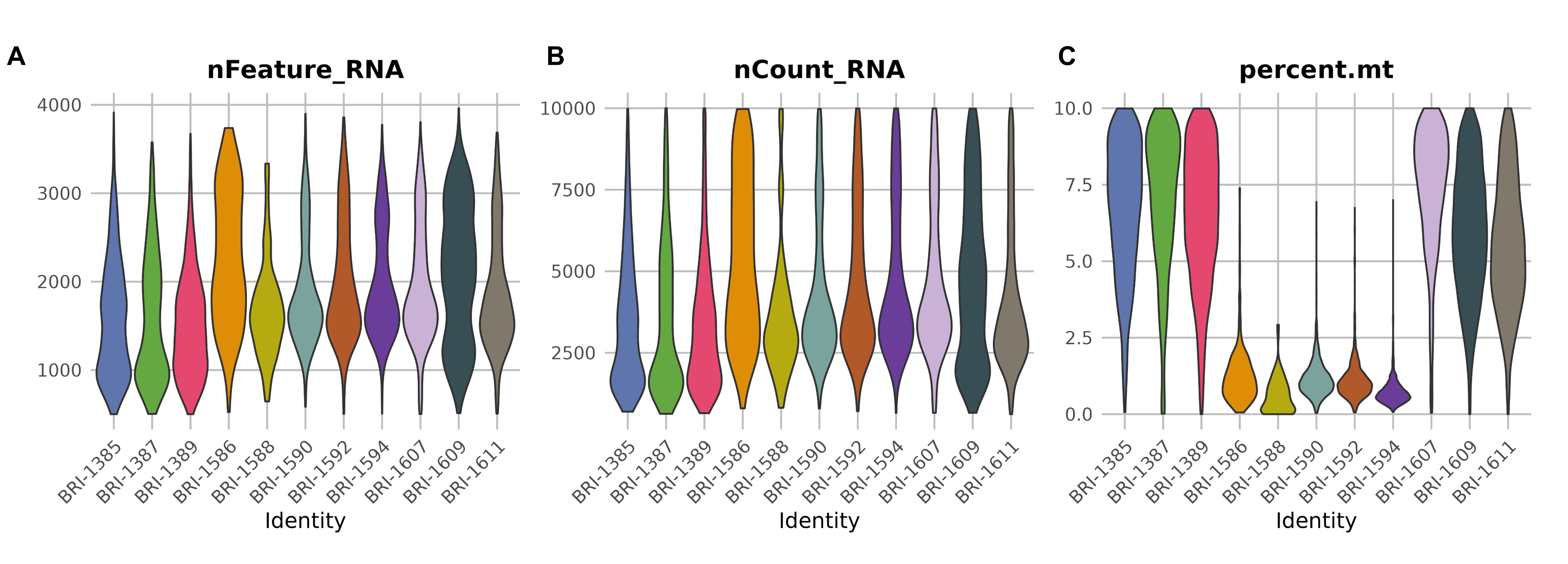

Supplement: Supplementary Figure S2 — Quality control of synovial single-cell RNA-seq dataset. (A) Violin plot of nFeature_RNA across samples. (B) Violin plot of nCount_RNA across samples. (C) Violin plot of mitochondrial gene percentage (percent.mt) across samples. These metrics collectively confirm the quality and comparability of synovial cell libraries. [file Image2.jpeg]

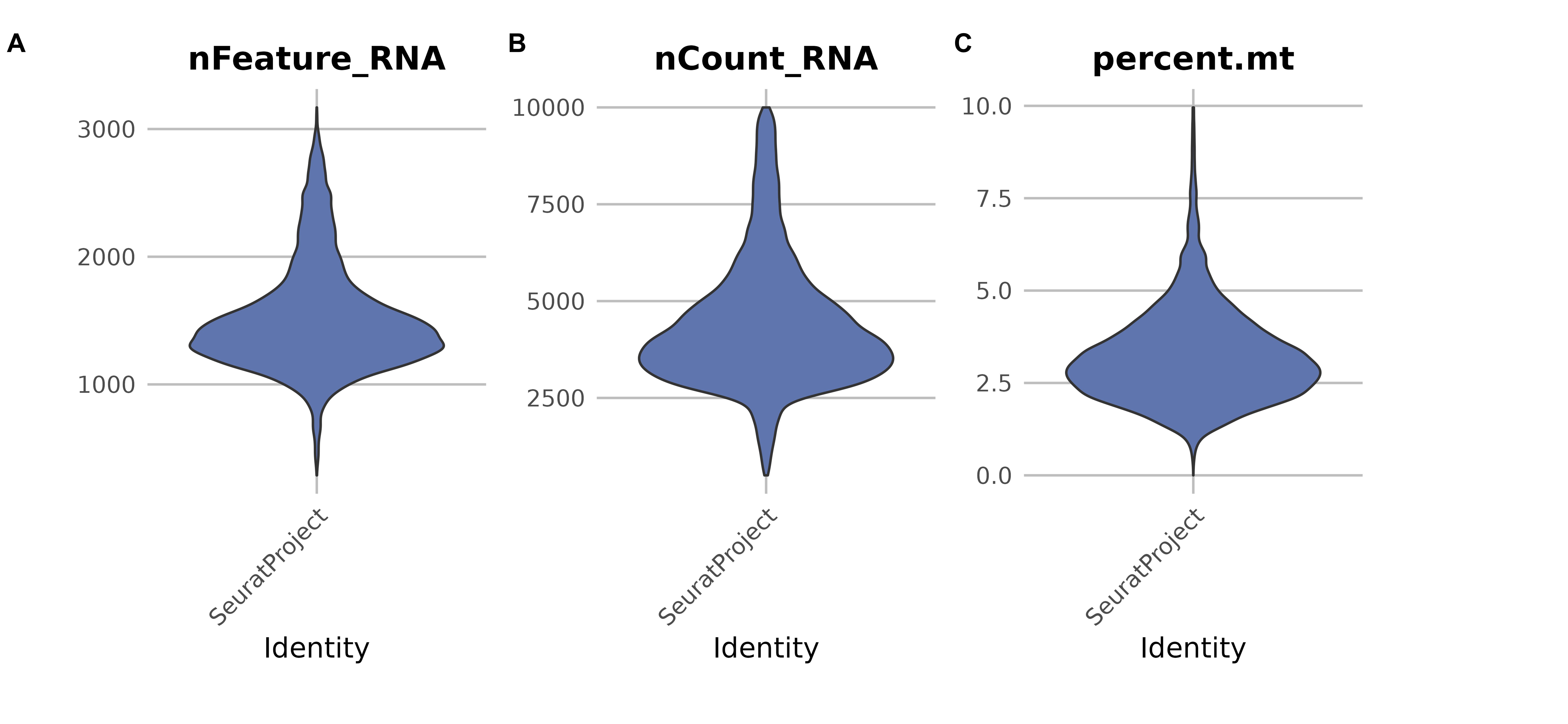

Supplement: Supplementary Figure S3 — Quality control of blood single-cell RNA-seq dataset. (A) Violin plot of nFeature_RNA. (B) Violin plot of nCount_RNA. (C) Violin plot of percent.mt. Quality distributions support reliable downstream single-cell analyses. [file Image3.jpeg]

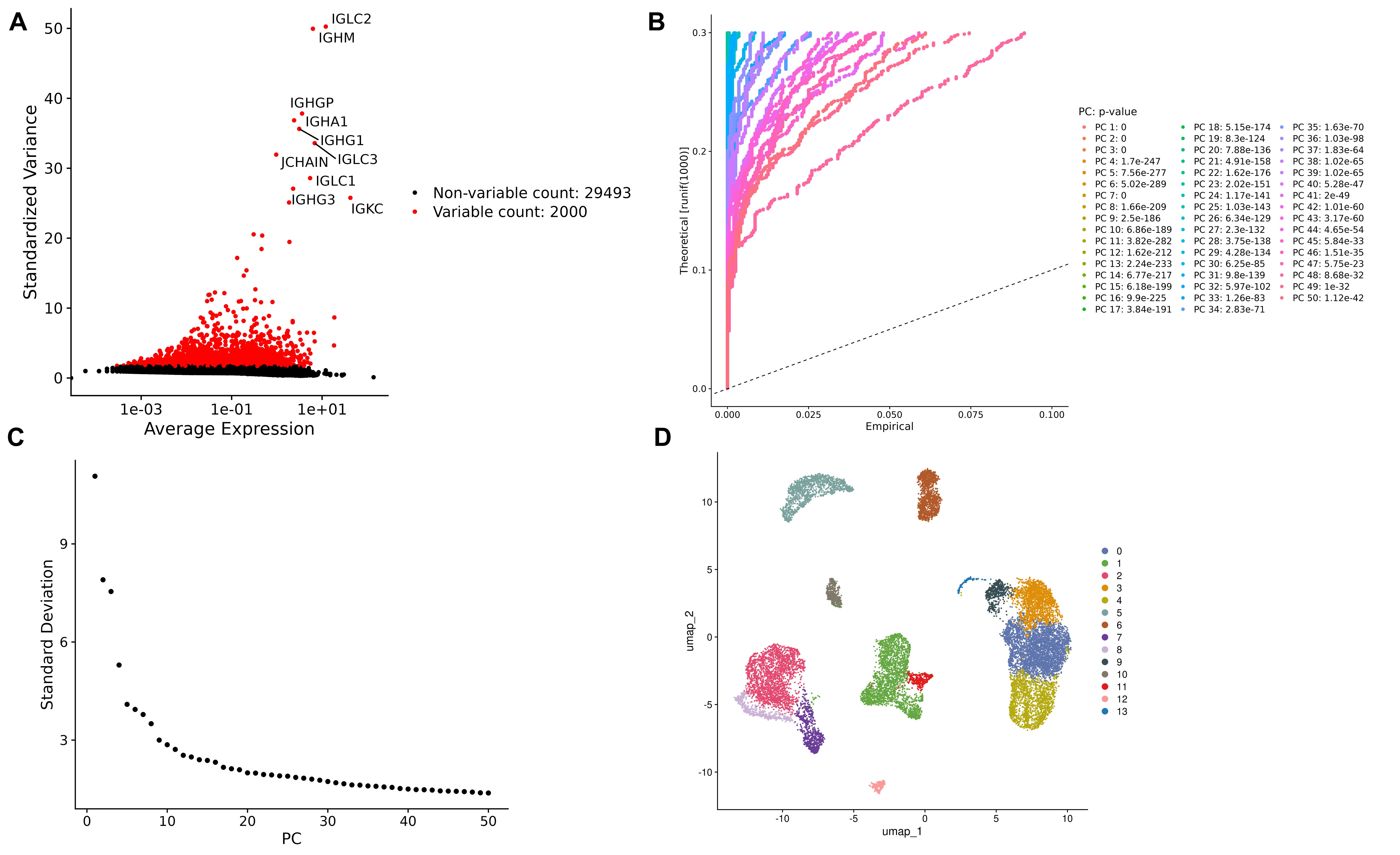

Supplement: Supplementary Figure S4 — Synovial single-cell variable gene detection and clustering. (A) Scatter plot of standardized variance vs. average expression, highlighting the top 2,000 variable genes. (B) JackStraw plot assessing statistical significance of PCs. (C) ElbowPlot showing standard deviation across PCs. (D) UMAP projection showing 14 clusters of synovial cells. [file Image4.jpeg]

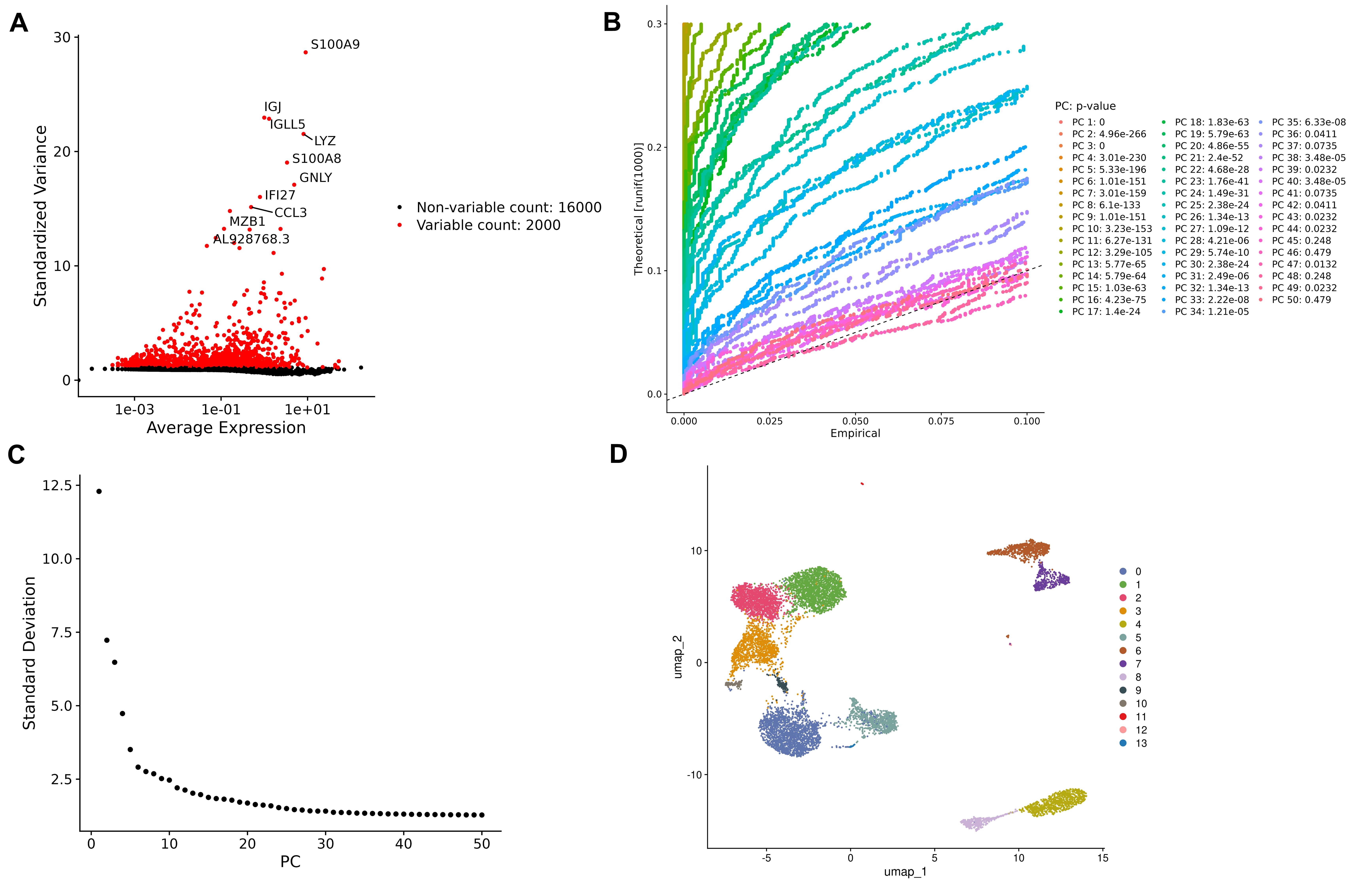

Supplement: Supplementary Figure S5 — Blood single-cell variable gene detection and clustering. (A) Highly variable gene selection in blood cells. (B) JackStraw plot showing significance of PCs. (C) ElbowPlot identifying major PCs. (D) UMAP projection identifying 14 blood cell clusters. [file Image5.jpeg]

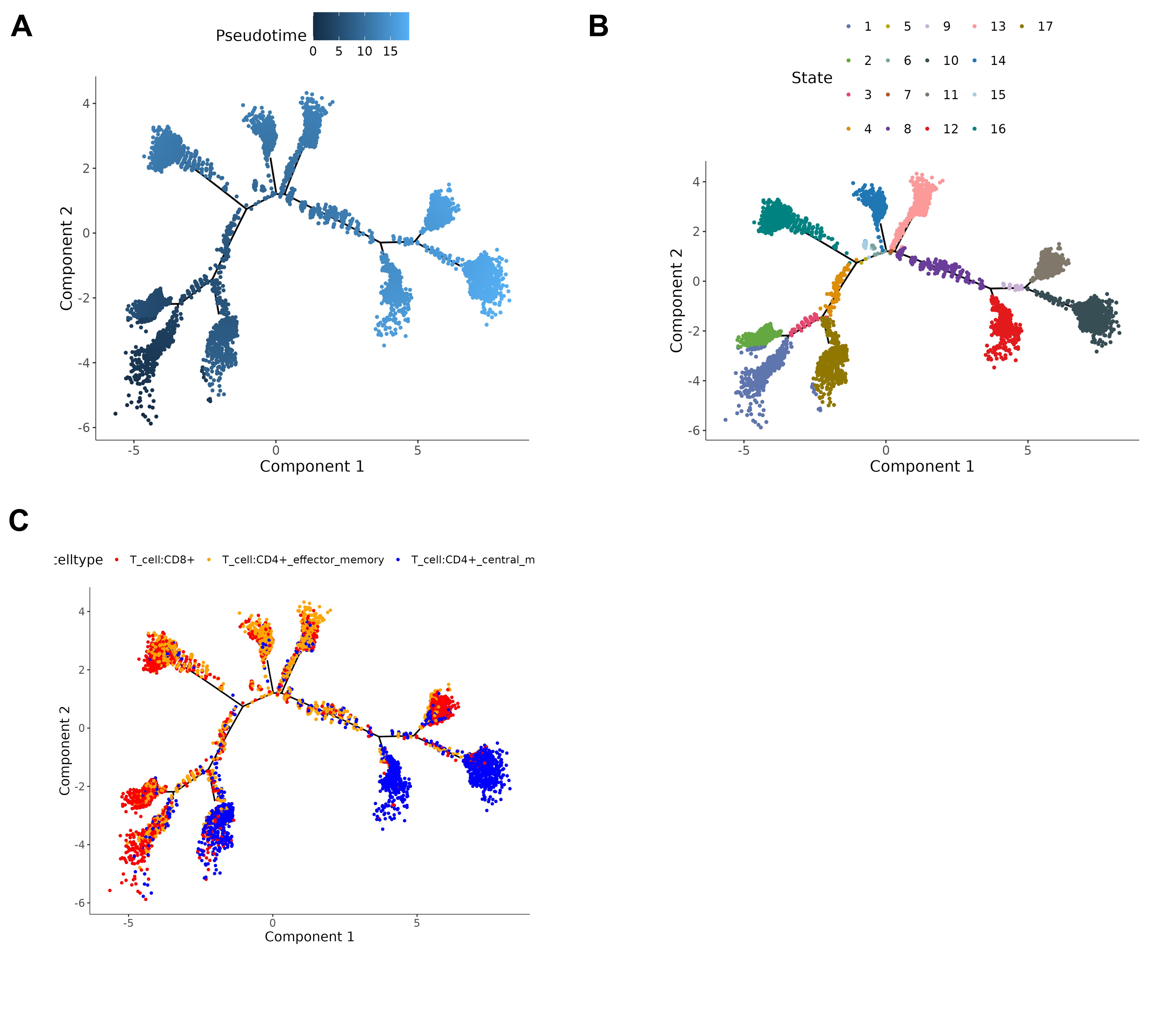

Supplement: Supplementary Figure S6 — Pseudotime analysis of synovial T cells. (A) Pseudotime trajectory showing differentiation continuum. (B) State assignment of 17 synovial T-cell states. (C) Distribution of T-cell subtypes (CD8+, CD4+ effector memory, CD4+ central memory) along trajectory. [file Image6.jpeg]

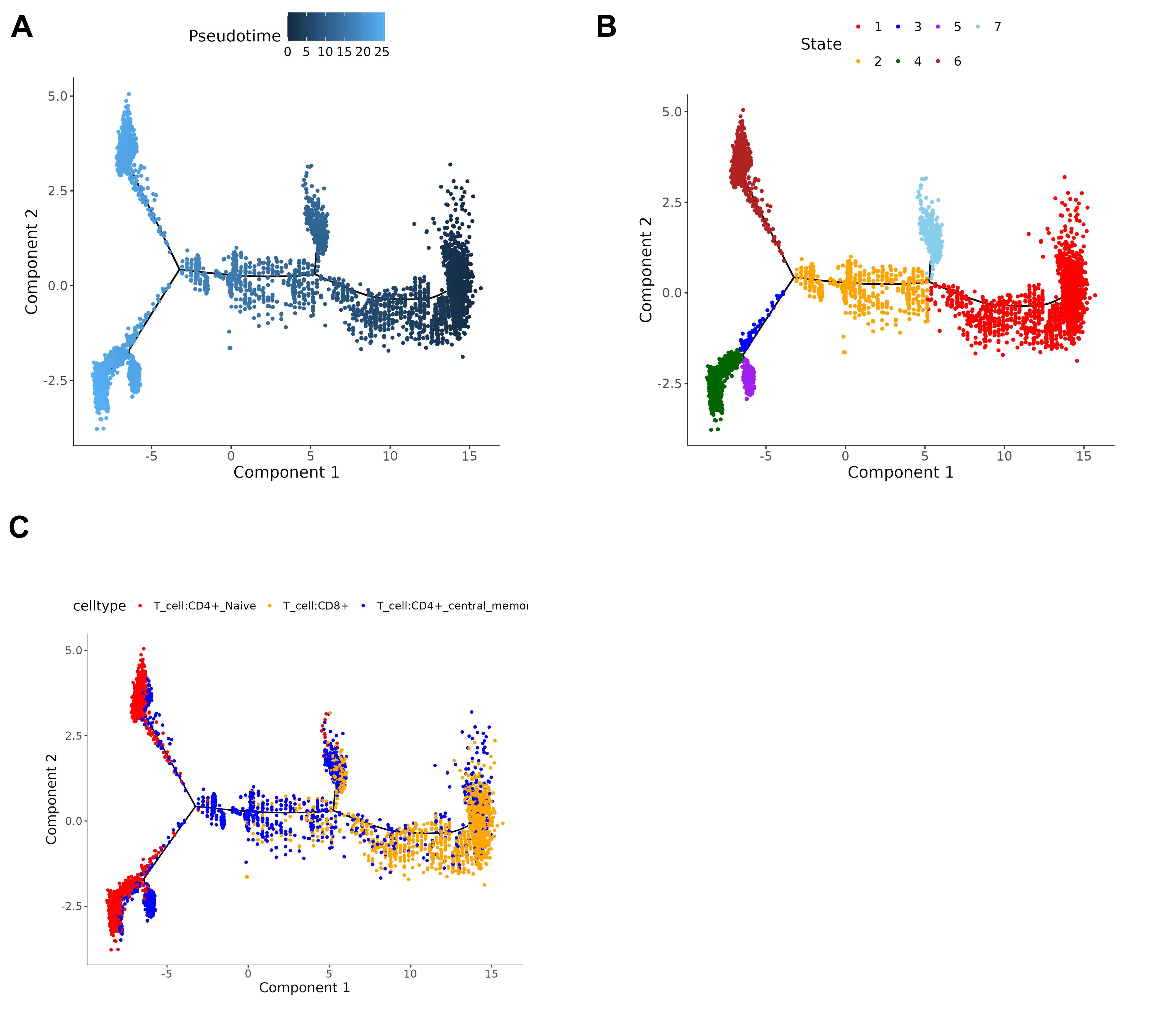

Supplement: Supplementary Figure S7 — Pseudotime analysis of blood T cells. (A) Pseudotime trajectory showing differentiation progression. (B) State assignment of 7 T-cell states. (C) Distribution of major blood T-cell subsets (CD4+ naïve, CD8+, CD4+ central memory). [file Image7.jpeg]

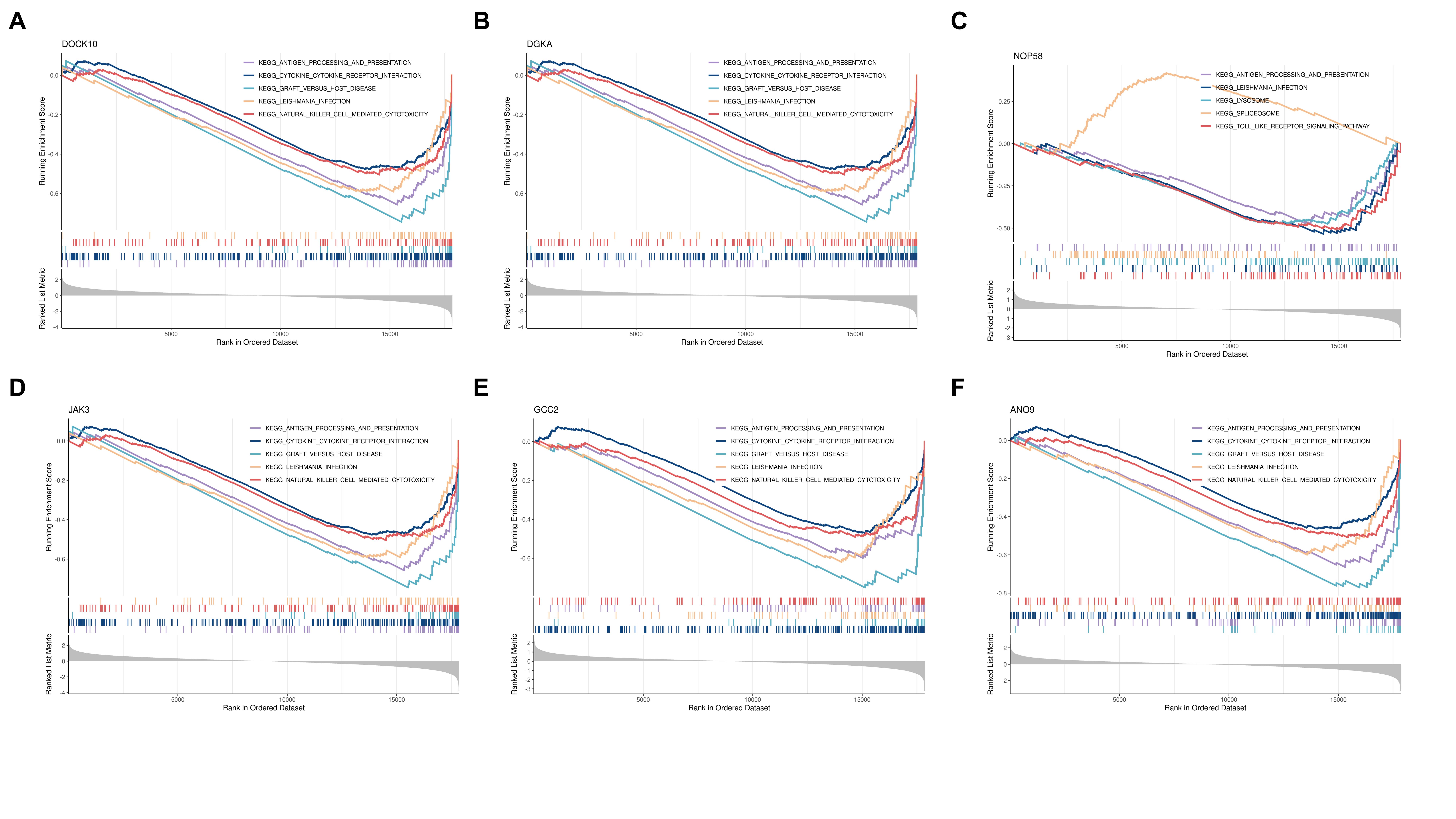

Supplement: Supplementary Figure S8 — GSEA of six key BS-TUGs. GSEA enrichment curves for: (A) DOCK10. (B) DGKA. (C) NOP58. (D) JAK3. (E) GCC2. (F) ANO9. Each panel shows the top KEGG pathways significantly enriched in high- or low-expression samples, including antigen processing and presentation, cytokine–cytokine receptor interaction, natural killer cell–mediated cytotoxicity, and spliceosome. [file Image8.jpeg]
