# Supplementary material for: Dual-omics analysis of key biomarkers in T cell ubiquitination of rheumatoid arthritis blood and synovial tissue, validated by two-sample Mendelian randomization and qPCR
Source: Front Immunol. 2026 Mar 2;17:1764990. doi: 10.3389/fimmu.2026.1764990 (PMC12989613; doi:10.3389/fimmu.2026.1764990)
Supplement: Supplementary file 11 [file Table2.docx]

Table S2. Summary of eQTL and GWAS Datasets Used for Mendelian Randomization Analysis

| **id** | **Sybmol/Feature** | **Year** | **population** | **sample.size** | **ncase** | **ncontrol** | **nsnp** |
| --- | --- | --- | --- | --- | --- | --- | --- |
| eqtl-a-ENSG00000055044 | NOP58 | 2018 | European | 31684 | - | - | 16293 |
| eqtl-a-ENSG00000185101 | ANO9 | 2018 | European | 26181 | - | - | 16517 |
| eqtl-a-ENSG00000105639 | JAK3 | 2018 | European | 31684 | - | - | 19043 |
| eqtl-a-ENSG00000135905 | DOCK10 | 2018 | European | 26609 | - | - | 17887 |
| eqtl-a-ENSG00000065357 | DGKA | 2018 | European | 30935 | - | - | 17580 |
| eqtl-a-ENSG00000135968 | GCC2 | 2018 | European | 31470 | - | - | 18493 |
| bbj-a-73 | Rheumatoid Arthritis | 2019 | European | 8383 | 5540 | 2843 | 8747962 |
